# Sulfiredoxin‐1 is a promising novel prognostic biomarker for hepatocellular carcinoma

**DOI:** 10.1002/cam4.3430

**Published:** 2020-09-21

**Authors:** Qian‐Wen Rao, Shi‐Long Zhang, Meng‐Zhou Guo, Fei‐Fei Yuan, Jia‐Lei Sun, Feng Qi, Li‐Shun Wang, Bi‐Wei Yang, Jing‐Lin Xia

**Affiliations:** ^1^ Minhang Branch Zhongshan Hospital Fudan University Shanghai China; ^2^ Liver Cancer Institute Zhongshan Hospital Fudan University Shanghai China; ^3^ Institute of Fudan‐Minhang Academic Health System Minhang Branch Zhongshan Hospital Fudan University Shanghai China

**Keywords:** Hepatocellular carcinoma, nomogram, prognosis, Sulfiredoxin‐1, survival analysis

## Abstract

Identifying novel prognostic biomarkers for hepatocellular carcinoma (HCC) and then, develop an effective individualized treatment strategy remain extremely warranted. The prognostic role of sulfiredoxin‐1(SRXN1), an antioxidant enzyme, remains unknown in HCC. This study aimed to explore the prognostic implications of SRXN1 in HCC patients after partial hepatectomy. The expression of SRXN1 in HCC and normal tissue were analyzed using the patients from the public databases and Zhongshan Hospital. The Cox regression, Kaplan‐Meier survival analysis, and time‐dependent receiver operating characteristic curves were performed to identify the predictive role of SRXN1 expression on HCC patients. A prognostic nomogram based on SRXN1 expression was constructed and validated to further confirm the predictive power of SRXN1 as a prognostic biomarker. Finally, functional enrichment analysis and protein‐protein interaction network analysis of SRXN1 and its associated genes were conducted. The results showed that SRXN1 was upregulated in HCC samples compared with the normal liver tissues. Patients with SRXN1 upregulation had shorter survival time. SRXN1 overexpression was significantly correlated with advanced clinicopathological parameters. The prognostic nomogram based on SRXN1 expression was proved to be more accurate than routine staging systems for the prediction of overall survival. Protein‐protein interaction network analysis demonstrated the first neighbor genes of SRXN1 mainly participated in response to oxidative stress. In brief, SRXN1 could be a prognostic biomarker for the management of HCC.

## Graphical abstract

In this work, we evaluated the prognostic power of SRXN1 for hepatocellular carcinoma using clinical specimens from both the public databases and Zhongshan Hospital. We also established a prognostic nomogram based on SRXN1 expression, which was proved to be more accurate than the routine staging systems for the prediction of overall survival. Finally, functional and pathway enrichment analysis and PPI network analysis of SRXN1 and its related genes were conducted, respectively, which may shed light on the mechanism of SRXN1 as an oncogene.

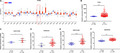

## 1 INTRODUCTION

Hepatocellular carcinoma (HCC), one of the most frequent malignant tumors in digestive system, is globally the fourth leading cause of cancer‐related death. The past decades have witnessed the evolvement of the treatment strategies of HCC. Despite great advances have been made in the molecular mechanisms and treatment strategies of HCC, the prognosis of patients, the majority of which are diagnosed at advanced disease stage, remain poor.^1^, ^2^, ^3^ Serum tumor markers can be used for diagnosis, prognostic judgment, and recurrence supervision of HCC noninvasively. Alpha‐fetoprotein (AFP) is the most common serum marker used for the diagnosis of HCC and the increased concentration of AFP usually indicates a poor prognosis. However, AFP lacks good sensitivity and specificity because it increases in merely 60%‐70% of HCC patients and some non‐HCC diseases as well.^3^, ^4^, ^5^ Currently, the Tumor Node Metastasis (TNM) and the Barcelona Clinic Liver Cancer (BCLC) staging system have been widely used in the staging systems and predicting the survival for HCC patients. Nevertheless, the two staging systems still need to be further refined due to the variation in outcomes of patients with end‐stage liver disease.^3^ Therefore, the accurate prognostic assessment is essential for proper management. Moreover, it is of great importance to identify new prognostic biomarkers that can predict the outcomes for HCC patients, and then develop an effective individualized treatment strategy.

Redox biology plays an irreplaceable role in maintaining biological physiological balance. Serving as signaling molecules regulating redox, reactive oxygen species (ROS) induce oxidative stress by damaging lipids, proteins, and DNA, which causes multiple human disorders, including cancer. A variety of internal and external antioxidant molecules are generated to protect the biological system from oxidative damages.^6^, ^7^, ^8^

Thiol‐based antioxidants occupy the majority of internal antioxidant molecules, in which sulfiredoxin‐1 (SRXN1) and peroxiredoxins earn a spot. SRXN1 is an antioxidant enzyme which protects the host cells from oxidative damages by catalyzing the reduction of hyperoxidized peroxiredoxins to the reduced form.^9^, ^10^ In addition, SRXN1 also participates in the deglutathionylation of some substrates in eukaryotes.^11^ Studies had shown that SRXN1 was overexpressed in multiple types of malignancies, including cervical cancer,^12^ colorectal cancer,^13^ skin cancer,^14^ lung cancer,^15^ and renal cell carcinoma.^16^ It had been demonstrated as a critical player in cell proliferation, migration, and metastasis,^6^ and its abnormal expression usually contributed to the poor survival.^15^, ^17^, ^18^, ^19^All these evidence revealed that SRXN1 functioned as an oncogene. Study had demonstrated SRXN1 and peroxiredoxin 1 (RRDX1) played a pivotal role in protecting the liver in ethanol‐fed mice against oxidative injury by eliminating ROS.^20^ However, the biological roles and prognostic values of SRXN1 in HCC remain unknown.

In our study, we intended to explore the prognostic implications of SRXN1 in HCC patients. The expression of SRXN1 in HCC and peritumoral tissue and Kaplan‐Meier survival analysis for SRXN1 expression in HCC were analyzed using the public databases. Then, the three quarters of tumor samples obtained from 269 HCC patients in Zhongshan Hospital were assigned to the training cohort and one quarter to the validation cohort. Immunohistochemistry (IHC) staining was performed to study the correlations between SRXN1 expression and clinicopathological characteristics, as well as patient survival. We also established a prognostic nomogram integrating SRXN1 expression and other independent prognostic factors to further confirm the predictive power of SRXN1 as a prognostic biomarker. Finally, protein‐protein interaction (PPI) network analysis of SRXN1 and its associated genes were conducted.

## 2 MATERIALS AND METHODS

### 2.1 Extraction of gene expression data the public databases

The gene expression of 421 HCC patients were acquired from the Cancer Genome Atlas (TCGA) database (http://www.cbioportal.org/data_sets.jsp). Gene expression of 371 HCC samples based on GSE101685, GSE62232, GSE121248, GSE45436, and GES73571 were downloaded from Gene Expression Omnibus (GEO) database (http://www.ncbi.nlm.nih.gov/geo/). These raw data from GEO database was successively processed by RMA background correction, log2 transformation and normalization using the R software (version 3.6.2) (http://www.r‐project.org/).

### 2.2 UALCAN

UALCAN (http://ualcan.path.uab.edu) is a free web resource for exploring transcriptome data in TCGA database. It provides graphs and plots of expression level and survival analysis of certain genes of interest. Additionally, UALCAN provides access to other databases, including HPRD, GeneCards, PubMed, TargetScan, The human protein atlas, DRUGBANK, Open Targets, and the GTEx to help researchers fully understand their selected genes.^21^ In our study, the expression of SRXN1 in tumor and normal tissue was comprehensively analyzed using the UALCAN.

### 2.3 The Kaplan‐Meier plotter analysis

The Kaplan‐Meier plotter (http://kmplot.com/analysis) is an open online database that can be used to investigate the prognostic role of a specific gene in TCGA database. The demographic, clinicopathologic characteristic, and several risk factors are also listed on the web page, enabling the researchers to further assess the prognostic value of a particular gene.^22^, ^23^ In our study, using the Kaplan‐Meier plotter, we investigated the impacts of SRXN1 mRNA expression on the overall survival (OS), relapse free survival (RFS), progression free survival (PFS), and disease‐specific survival (DSS) among the HCC patients using the upper tertile as the cutoff value.

### 2.4 Patients and follow‐up

A total of 269 HCC patients who underwent surgical resection from Zhongshan Hospital, Fudan University were enrolled in this retrospective study between January 2005 and February 2012. Patient inclusion and exclusion criteria are as below: (a) Postoperative pathology confirmed as HCC; (b) The liver function of all patients was Child‐Pugh class A before partial hepatectomy; (c) Patients with mixed liver cancer were excluded; (d) Patients with incomplete clinical information and follow‐up data were excluded.

The clinicopathological information provided were as follows: gender, age, AFP, hepatitis B virus surface antigen (HBsAg), cirrhosis, tumor number, tumor size, microvascular invasion, tumor grade, and tumor stage. Tumor stage was evaluated by both the American Joint Committee on the TNM staging system (8th edition) and BCLC staging system. TNM stage III + IV or BCLC stage B + C were defined as advanced stage. OS was calculated as the interval from the date of partial hepatectomy to the last follow‐up or the day of death. The deadline for follow‐up data were 13 September 2017, with the median OS of 64.8 months (ranging from 0.5 to 110.0 months). All the tumor and paracancerous tissue samples were obtained based on the informed consent of the patients and our study was approved by the ethics committee of Zhongshan Hospital.

### 2.5 Immunohistochemistry

IHC was conducted on tissue sections based on the standard streptavidin‐peroxidase method. Paraffin wax‐embedded sections were deparaffinized in xylene and hydrated in descending series of ethanol after baking at 65°C for 2 hours. Antigen retrieval was finished with 0.01M sodium citrate buffer solution (pH6.0), and then, incubation overnight at 4°C with SRXN1 antibody (1:100, Proteintech, 14273‐1‐AP) was performed. Tissue sections were incubated with the horseradish peroxidase‐conjugated secondary antibody at room temperature for 30min. Antigen distribution in tumor was visualized under a light microscopy in the diaminobenzidine solution and hematoxylin. Taking into account the staining intensity (0, negative; 1, weak; 2, intermediate; 3, strong) and the percentage of positive cells expressed by SRXN1 (0,0% positive cells; 1, 1%‐25% positive cells; 2, 26%‐50% positive cells; 3, 51%‐75% positive cells; and 4, 76%‐100% positive cells), two independent pathologists who were unaware of clinical data implemented the evaluation of SRXN1 expression using a semi‐quantitative histological score system. Comprehensive score was multiplied by the staining intensity score and staining proportion score. Finally, SRXN1 expression level was divided into the following four levels: 0, negative; 1‐4, weak; 6‐8, moderate; and 9‐12, strong.

### 2.6 Quantitative Real‐Time PCR

Total RNA from the tumor and paracancerous samples were extracted using TRIzol (Invitrogen Life Technologies, USA). β‐actin were used for the housekeeping gene. SRXN1 primers: forward primer 5′‐CAGGGAGGTGACTACTTCTACTC‐3′, reverse primer 5′‐CAGGTACACCCTTAGGTCTGA‐3′; β‐actin primers: forward primer 5′‐ CCACGAAACTACCTTCAACTCC‐3′, reverse primer 5′‐ GTGATCTCCTTCTGCATCCTGT‐3′.

### 2.7 Western blotting analysis

All the samples were homogenized with RIPA lysis buffer (Beyotime, China). The protein concentrations of the samples were determined by BCA kit (Beyotime, China). Equal amounts of protein samples were separated by electrophoresis on 12% SDS‐PAGE and then, were transferred onto polyvinylidene fluoride membranes. Membranes were incubated with primary antibodies (anti‐SRXN1, 1:1000, Proteintech, 14273‐1‐AP) at 4°C overnight after beening blocked. The horseradish peroxidase‐conjugated secondary antibodies were used to incubate the membranes. The protein bands were visualized and quantified using a chemiluminescent substrate ECL kit and ImageJ software, respectively.

### 2.8 Gene ontology (GO) and pathway enrichment analysis

The Database for Annotation, Visualization, and Integrated Discovery (DAVID; https://david.ncifcrf.gov/) is an online tool used for functional annotation analysis, which enables researchers to comprehend the biological meaning of listed genes.^24^, ^25^ GO and Kyoto Encyclopedia of Genes and Genomes (KEGG) pathway enrichment analysis of SRXN1 and its associated genes were performed by DAVID.

### 2.9 PPI network construction

PPI network was constructed by the Search Tool for the Retrieval of Interacting Genes (STRING; https://stringdb.org/) and visualized by the software Cytoscape (version.3.7.0). To evaluate the relationship between SRXN1 and its related genes, we uploaded all the genes to STRING and set minimum required interaction score as medium confidence (0.4).

### 2.10 Statistical analysis

Statistical analyses were performed using SPSS 22.0 software. Categorical variables were analyzed by the χ2 test or Fisher's exact test and continuous variables were compared using the t test. The OS differences between the two groups were evaluated by the Kaplan‐Meier method and compared with the log‐rank test. Univariate and multivariate Cox proportional hazard regression analyses were performed to identify significant factors associated with OS.

A nomogram was developed through integrating significantly independent prognostic factors derived from the multivariate Cox analysis using “rms” package. The performance of the nomogram was evaluated and validation by the training cohort and the validation cohort, respectively. The predictive accuracy of this nomogram was assessed with the Area Under the Curve (AUC) values in the receiver operating characteristic (ROC) curve and Harrell's concordance index (C‐index) and was evaluated by comparing the predicted survival with the observed survival. The larger the AUC, the larger the C‐index, the closer the observed and predicted survival, and the more accurate the prognostic nomogram was. Statistical analysis of different AUCs was performed with the MedCalc statistical software (version 19.3.1). *P* < .05 was considered statistically significant.

## 3 RESULTS

### 3.1 Transcriptional expression of SRXN1 in HCC patients

**Figure 1:**
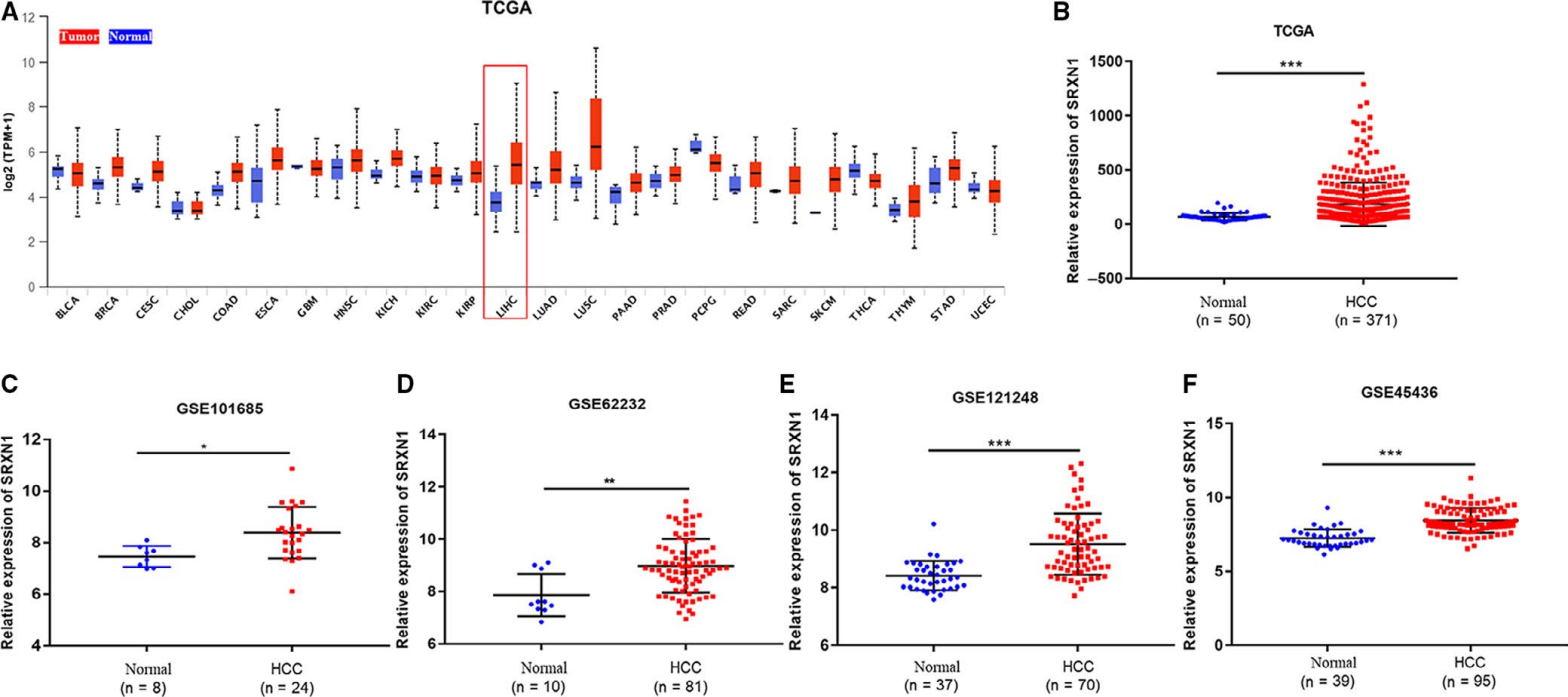
The mRNA expression of SRXN1 in tumor tissues and normal tissues based on the public databases. (A) SRXN1 expression in various tumor types based on the UALCAN database; (B) SRXN1 expression in HCC based on the TCGA database; (C‐F) SRXN1 expression in HCC based on the GEO database. *, *P* < .05; **, *P* < .01; ***, *P* < .001. TCGA, the cancer genome atlas; GEO, Gene expression omnibus

**Figure 2:**
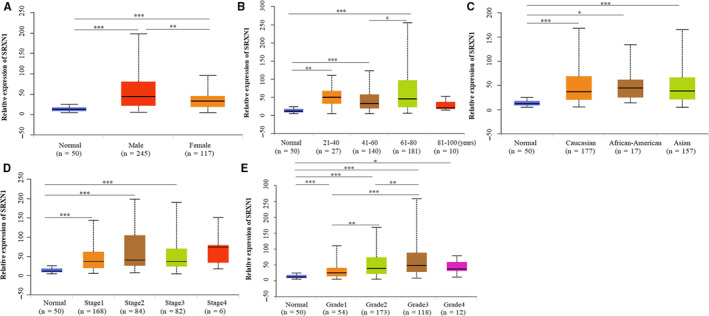
Subgroup analysis of SRXN1 expression in HCC based on TCGA database. All HCC patients were stratified according to gender(A), Age(B), Race(C), Stage(D), and grade(E). *, *P* < .05; **, *P* < .01; ***, *P* < .001. TCGA, The cancer genome atlas

To filter out prognostic genes for HCC, we first screened out 787 common differential genes (DEGs) of TCGA, GSE121248, and GSE45436.Then 56 out of 787 DEGs were found to participate in the regulation of redox biology using STRING database. Six genes including SRXN1 whose prognostic value for HCC remain unclear were filtered out. In addition, the expression of these prognostic genes was further verified both in GSE101685 and GSE62232 (Figure S1). As a result, we investigate the role of SRXN1 in HCC. The mRNA expression of SRXN1 in 24 types of cancers and normal tissues samples was visualized by the UALCAN website. As showed in Figure 1A, the transcriptional expression of SRXN1 in cancer tissues were significantly higher than that of normal tissues. We further investigated the expression of SRXN1 in HCC patients and normal liver tissues. SRXN1 was overexpressed in HCC tissues compared with normal liver tissues in both TCGA and GEO databases (Figure 1B‐1F). Next, SRXN1 expression in different subgroups of HCC patients from TCGA database was further analyzed based on gender, age, ethnicity, individual HCC cancer stages, and tumor grade. The results showed SRXN1 expression was higher in separate subgroups of HCC patients compared with a healthy population, which indicated the high expression of SRXN1 was universal in HCC regardless of gender, age, race, stage, and grade (Figure 2).

### 3.2 Kaplan‐Meier curves for SRXN1 expression in HCC based on TCGA database

**Figure 3:**
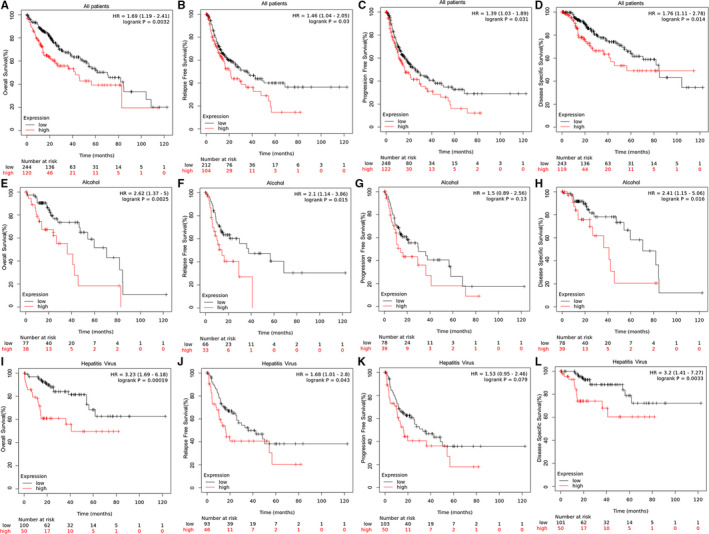
Kaplan‐Meier curves of HCC patients from TCGA database according to SRXN1 expression. (A‐D) All patients; (E‐H) Patients with alcohol consumption; (I‐L) Patients with hepatitis virus infection. P value was calculated using the log‐rank test and was described in the upper right corner of each graph. *P* < .05 is considered as statistically significant. TCGA, The Cancer Genome Atlas

In order to examine whether SRXN1 was associated with worse prognosis of HCC patients, we investigated the impacts of SRXN1 mRNA expression on OS, RFS, PFS, and DSS among HCC patients from TCGA database using the Kaplan‐Meier plotter. The results showed us that elevated SRXN1 expression was significantly correlated with shorter OS (*P* = .0032), RFS (*P* = .03), PFS (*P* = .031), and DSS (*P* = .014) (Figure 3A‐D). Considering both alcohol and hepatitis virus were risk factors for HCC, we appraised the association between SRXN1 expression and the survival of HCC patients with alcohol consumption and hepatitis virus infection. Surprisingly, the high expression of SRXN1 revealed good power of predicting outcomes of patients with alcohol consumption and hepatitis virus infection (Figure 3E‐L).

**Figure 4:**
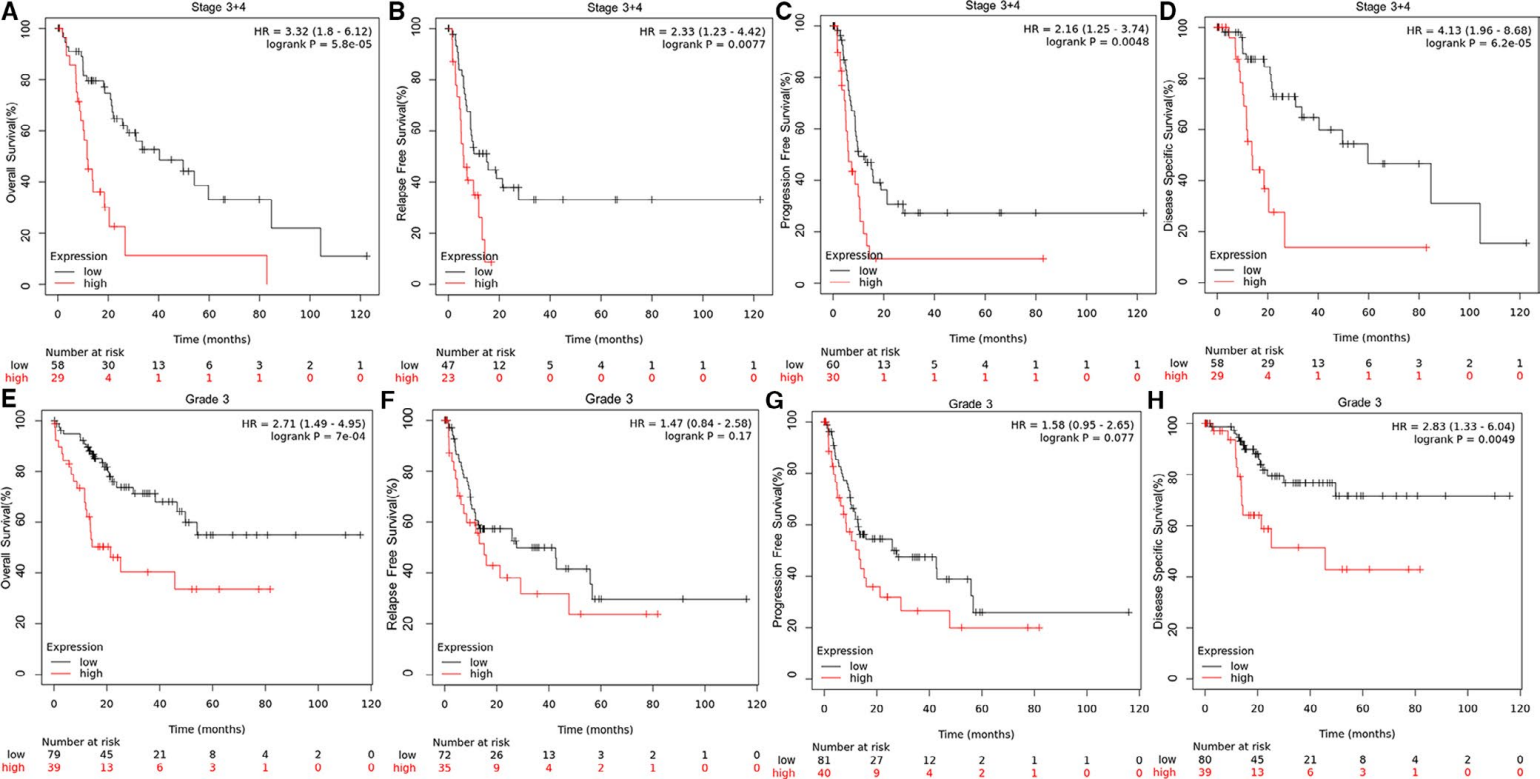
Kaplan‐Meier curves of HCC patients with advanced clinicopathological parameters from TCGA database according to SRXN1 expression. (A‐D) Patients with advanced state; (E‐H) Patients with advanced grade. P value was calculated using the log‐rank test and was described in the upper right corner of each graph. *P* < .05 is considered as statistically significant. TCGA, The Cancer Genome Atlas

The expression of SRXN1 seemed to be higher in HCC patients with advanced clinicopathological parameters according to Figure 2D and E. Hence, we assessed the prognostic value of SRXN1 expression on poorly differentiated and advanced state HCC patients. Surprisingly, the high expression of SRXN1 revealed good power of predicting outcomes of patients with poorly differentiated and advanced state (Figure 4).

### 3.3 Correlation between SRXN1 expression and clinicopathological characteristics of HCC patients

**Figure 5:**
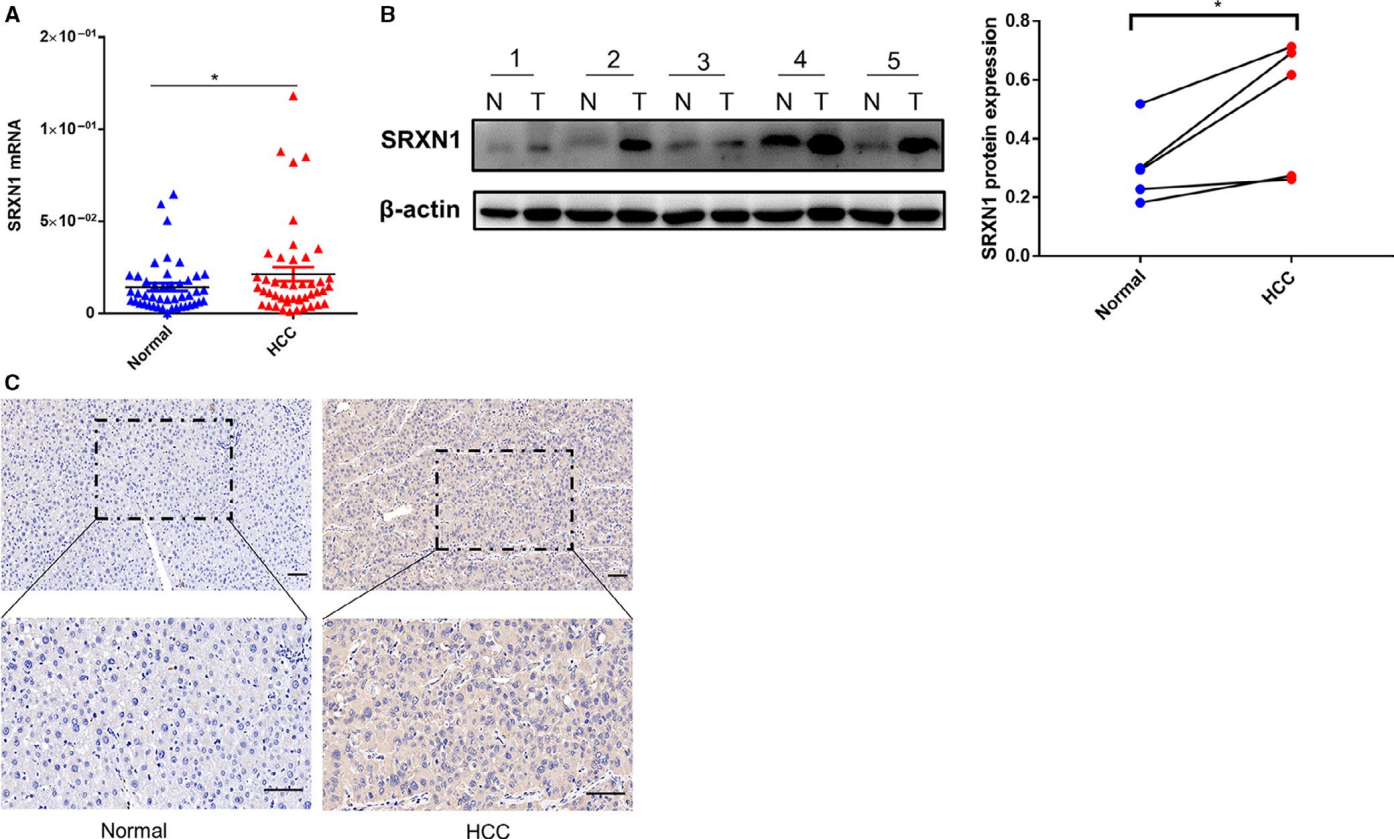
The mRNA and protein expression of SRXN1 in tumor tissues and normal tissues. (A) The mRNA expression of SRXN1 in 45 paired tumor and normal tissues; (B) The protein expression of SRXN1 in 5 paired tumor and normal tissues. T, tumor samples; N, paired normal samples;(C) Representative immunohistochemical staining of SRXN1 in HCC and normal tissue. Images are presented at × 200 (upper) and × 400 (lower) magnification. Scale bar: 50µm. *, *P* < .05

To confirm the predictive power of SRXN1 as a biomarker, we enrolled 269 HCC patients who had received partial hepatectomy from Zhongshan Hospital in our study. Both SRXN1 mRNA and protein expression were elevated in tumor samples compared to the paired normal samples (Figure 5A‐B). Then we divided the 269 HCC patients into the training cohort and the validation cohort in a ratio of 3:1 randomly. That is to say, the study involved 205 patients in the training cohort and 64 patients in the validation cohort. Afterwards, we further explored the expression of SRXN1 in HCC patients by IHC. Figure 5C showed SRXN1 mainly existed in cytoplasm and was overexpressed in tumor compared to the normal tissue. IHC analysis presented, among training cohort, 51 (24.88%) samples expressed SRXN1 strongly, 65 (31.71%) samples expressed SRXN1 moderately and only 32 (15.61%) samples expressed SRXN1 negatively, while in the validation cohort,16 (25%) samples expressed SRXN1 strongly, 18 (28.13%) samples expressed SRXN1 moderately and only four (6.25%) samples expressed SRXN1 negatively (Table S1). Furthermore, we defined the IHC score of 6 or above as high expression of SRXN1, and the score of less than 6 as low expression of SRXN1 according to the previous studies.^26^, ^27^ A total of 116 (56.59%) patients of the training cohort had SRXN1 upregulation while 89 (43.41%) patients had SRXN1 downregulation. A total of 34 (53.13%) patients had high SRXN1 expression while 30 (46.89%) patients had low SRXN1 expression in the validation cohort (Table S1). Table 1 described the clinicopathological characteristics of patients in the training cohort and validation cohort. Then the correlation between tumor SRXN1 level and clinicopathological characteristics was investigated (Table 2). The results depicted that SRXN1 level was significantly associated with tumor size (*P* = .006), TNM stage (*P* = .025), and BCLC stage (*P* < .001) in the training cohort. However, SRXN1 level was merely associated with age, cirrhosis, and HBsAg in the validation cohort, due to the small sample size.

**Table 1:** Demographics and clinic characteristics of patients with HCC

Characteristics	Training cohort (N = 205)	Validation cohort (N = 64)	*P* value
	Patients Number (%)	Patients Number (%)	
Gender			
Female	27(13.17)	11(17.19)	.231
Male	178(86.83)	53(82.81)	
Age, years			
<50	63(30.73)	24(37.50)	.312
≥50	142(69.27)	40(62.50)	
Cirrhosis			
Negative	84(40.98)	18(28.13)	.064
Positive	121(59.02)	46(71.87)	
HBsAg			
Negative	39(19.02)	12(18.75)	.961
Positive	166(80.98)	52(81.25)	
AFP, ng/mL			
<400	155(75.61)	36(56.25)	.003
≥400	50(24.39)	28(43.75)	
Tumor number			
Single	168(81.95)	58(90.63)	.098
Multiple	37(18.05)	6(9.37)	
Tumor size, cm			
≤5	129(62.93)	31(48.44)	.039
>5	76(37.07)	33(51.56)	
Microvascular invasion			
Absent	141(68.78)	43(67.19)	.811
Present	64(31.22)	21(32.81)	
Grade			
I‐II	146(71.22)	47(73.44)	.731
III‐IV	59(28.78)	17(26.56)	
BCLC stage			
A	124(60.49)	25(39.06)	.003
B + C	81(39.51)	39(60.94)	
TNM stage			
I + II	179(87.32)	53(82.81)	.361
III + IV	26(12.68)	11(17.19)	

Abbreviations: AFP, αfetoprotein; BCLC, Barcelona Clinic Liver Cancer; HBsAg, hepatitis B surface antigen; TNM, Tumor‐Nodes‐Metastases.

**Table 2:** Correlation between SRXN1 expression and clinicopathological characteristics of HCC patients in the training cohort and the validation cohort

Characteristics	Training cohort		*P* value	Validation cohort		*P* value
	SRXN1 level			SRXN1 level		
	Low	High		Low	High	
**All patients**	89	116		30	34	
Gender						
Female	13	14	0.594	5	6	0.917
Male	76	102		25	28	
Age, years						
<50	29	34	0.615	7	17	0.028*
≥50	60	82		23	17	
Cirrhosis						
Negative	39	45	0.468	12	6	0.047*
Positive	50	71		18	28	
HBsAg						
Negative	17	22	0.980	9	3	0.030*
Positive	72	94		21	31	
AFP, ng/mL						
<400	69	86	0.575	18	18	0.570
≥400	20	30		12	16	
Tumor number						
Single	72	96	0.731	27	31	0.872
Multiple	17	20		3	3	
Tumor size, cm						
≤5	71	72	0.006**	16	17	0.790
>5	18	44		14	17	
Microvascular invasion						
Absent	63	78	0.587	21	22	0.653
Present	26	38		9	12	
Grade						
I‐II	62	84	0.666	22	25	0.986
III‐IV	27	32		8	9	
BCLC stage						
A	67	57	<0.001***	13	12	0.511
B + C	22	59		17	22	
TNM stage						
I + II	83	96	0.025*	27	26	0.152
III + IV	6	20		3	8	

Abbreviation: AFP, αfetoprotein; BCLC, Barcelona Clinic Liver Cancer; HBsAg, hepatitis B surface antigen; TNM, Tumor‐Nodes‐Metastases. Statistical analyses were conducted with Pearson χ2 tests.

^*^ *P* < .05

^**^ *P* < .01

^***^ *P* < .001.

### 3.4 Overexpression of SRXN1 was an independent prognostic factor associated with worse survival

To test whether SRXN1 performed a clinically independent prognostic value in HCC patients, univariate and multivariate Cox regression analyses were used in the training cohort. Univariate analysis demonstrated that the high expression of SRXN1 was significantly correlated with a poor OS (hazard ratio ^[HR]^:1.958; 95% confidence interval [95% CI]:1.242‐3.089; *P* = .004). In addition, AFP, tumor size, tumor number, cirrhosis, HBsAg, microvascular invasion, BCLC stage, and TNM stage were all significantly associated with OS (Table 3). All significant variables found above were then included in the multivariate analysis. Just as shown in Table 3, SRXN1 remained as an independent predictor of OS in HCC patients, with a HR of 1.945(95%CI:1.197‐3.159, *P* = .007), as well as cirrhosis (HR:1.866; 95%CI:1.126‐3.091; *P* = .015), HBsAg (HR:2.574; 95%CI:1.248‐5.309; *P* = .010), and TNM stage (HR:2.499; 95%CI:1.287‐4.855; *P* = .007) after adjustment confounding variables.

**Table 3:** Univariate and multivariate analysis of the training cohort

Variables	Univariate analysis		Multivariate analysis	
	HR (95% CI)	*P*	HR (95% CI)	*P*
Gender	0.984(0.508‐1.907)	.962	‐	‐
Male vs Female				
Age	1.326(0.808‐2.175)	.264	‐	‐
≥50 vs <50				
SRXN1 expression High vs Low	1.958(1.242‐3.089)	.004^**^	1.945(1.197‐3.159)	.007^**^
AFP	1.952(1.219‐3.125)	.005^**^	1.307(0.756‐2.260)	.338
≥400 vs <400				
Tumor size	1.861(1.196‐2.897)	.006^**^	1.269(0.588‐2.738)	.545
>5 vs ≤ 5				
Tumor number	1.769(1.077‐2.906)	.024*	1.745(0.998‐3.049)	.051
Multiple vs Single				
Cirrhosis	1.818(1.123‐2.944)	.015*	1.866(1.126‐3.091)	.015*
Positive vs Negative				
HBsAg	2.267(1.133‐4.536)	.021*	2.574(1.248‐5.309)	.010*
Positive vs Negative				
Microvascular invasion	1.604(1.028‐2.504)	.037*	1.413(0.852‐2.342)	.180
Present vs Absent				
Grade	1.232(0.766‐1.984)	.389	‐	‐
III‐IV vs I‐II				
BCLC stage	2.162(1.405‐3.328)	*P* < .001^***^	1.313(0.626‐2.755)	.471
B + C vs A				
TNM stage	3.794(2.232‐6.448)	*P* < .001^***^	2.499(1.287‐4.855)	.007^**^
III + IV vs I + II				

Abbreviation: AFP, αfetoprotein; BCLC, Barcelona Clinic Liver Cancer; CI, confidential interval; HBsAg, hepatitis B surface antigen; TNM, Tumor‐Nodes‐Metastases; HR, hazard ratio.

*P < .05

^**^ *P* < .01

^***^ *P* < .001.

**Figure 6:**
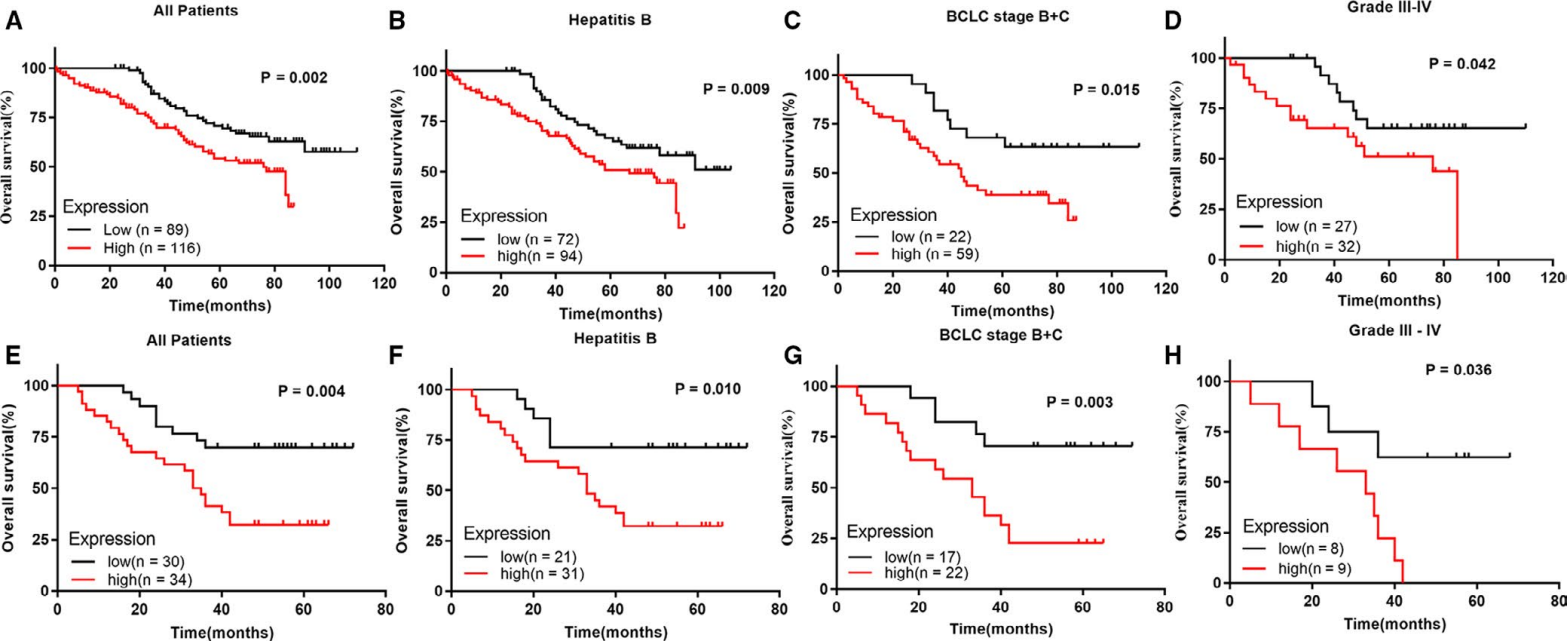
Kaplan‐Meier survival analysis of HCC patients according to SRXN1 expression in the training cohort and validation cohort, respectively. (A) All HCC patients from the training cohort; (B) HCC patients with Hepatitis B infection from the training cohort; (C) HCC patients with BCLC stage B + C from the training cohort; (D) HCC patients with grade III‐IV from the training cohort;(E) All HCC patients from the validation cohort; (F) HCC patients with Hepatitis B infection from the validation cohort; (G) HCC patients with BCLC stage B + C from the validation cohort; (H) HCC patients with grade III‐IV from the validation cohort. P value was calculated using the log‐rank test and was described in the upper right corner of each graph. *P* < .05 is considered as statistically significant. BCLC, Barcelona Clinic Liver Cancer staging system

Next, Kaplan‐Meier survival analysis showed a worse prognosis in patients with SRXN1 upregulation from the training cohort than those with SRXN1 downregulation (*P* = .002), which was further confirmed in the validation cohort (*P* = .004) (Figure 6A and E). For the training cohort, the median OS and the 5‐year OS rates were 63.0 months and 54.35%, respectively, in the upregulated SRXN1 subgroup, which were lower than those in the downregulated SRXN1 subgroup (72.7 months and 70.94%, respectively). For the validation cohort, the median OS and the probabilities of OS at 5 years (45.6 months and 32.35%, respectively) in upregulated SRXN1 subgroup were inferior with those in the downregulated SRXN1 subgroup (55.8 months and 70.00%, respectively) (Figure 6A and E). Moreover, the SRXN1 overexpression brought HCC patients who infected with hepatitis B worse outcome in the training cohort, and the consistent phenomenon was observed in patients of the validation cohort (Figure 6B and F). The staging of HCC patients was determined on BCLC and TNM staging system. Surprisingly, the high expression of SRXN1 also revealed good power of predicting outcomes of patients with poorly differentiated and advanced state not only in the training cohort, but also in the validation cohort (Figure 6C, D, G, and H). However, the abnormal expression of SRXN1 showed no significant effect on the OS of patients with TNM stage III‐IV due to the limited sample size both in the training cohort and validation cohort. Data were not shown. To assess the performance of SRXN1 as a biomarker for HCC, we examined the AUC values in the time‐dependent ROC curves. The 1‐year, 3‐year, and 5‐year AUC values showed SRXN1 had a good biomarker performance for HCC patients, especially in patients with poorly differentiated, advanced state or hepatitis B in the training cohort, and the results of the validated group confirmed the conclusion (Figure S2).

### 3.5 Prognostic nomogram for OS based on SRXN1 expression

**Figure 7:**
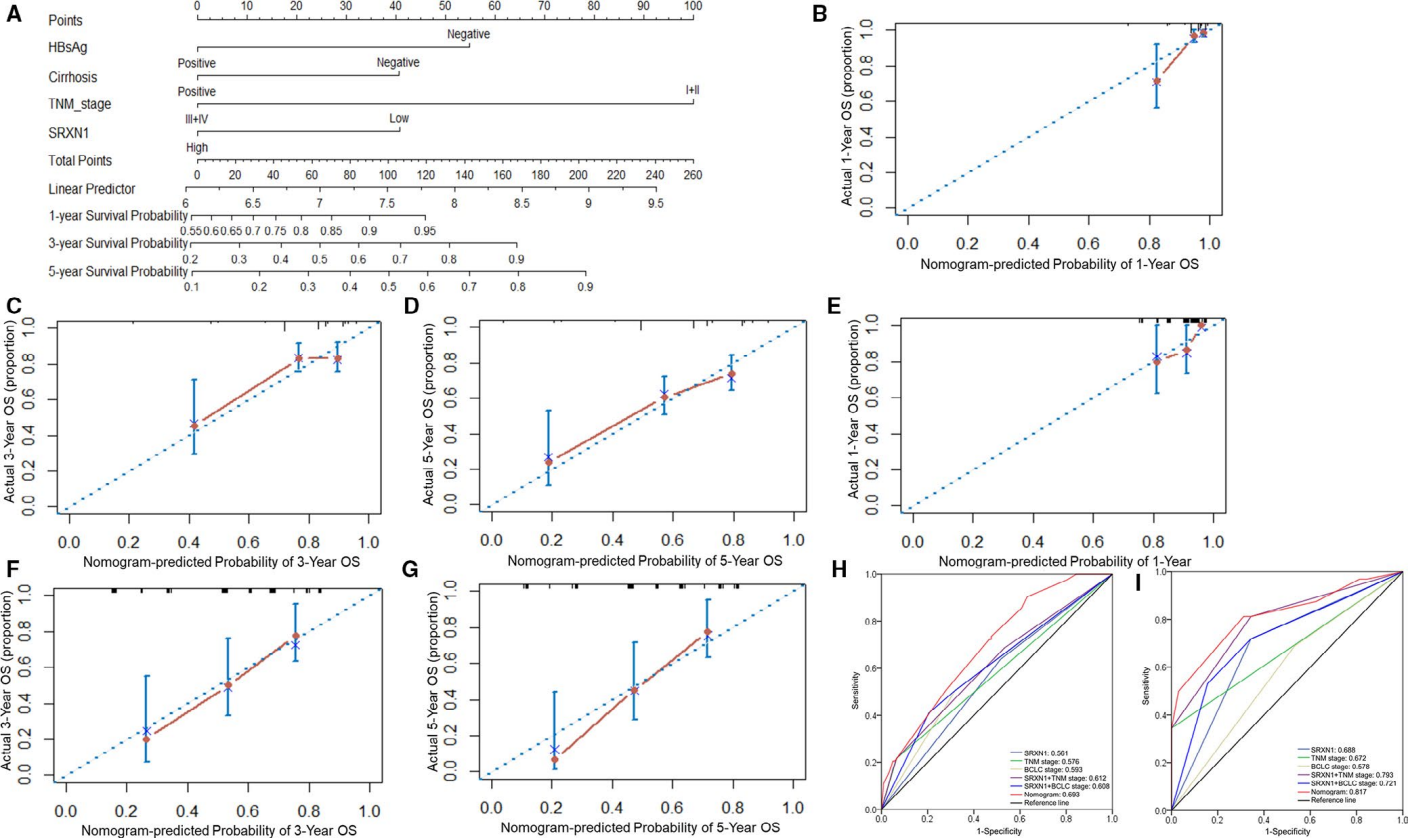
Nomogram, calibration plots and ROC curves for the prediction of the outcomes of HCC patients. (A) Nomogram for the prediction of OS at 1, 3, and 5 years; (B‐D) The calibration plots for predicting 1‐year, 3‐year, and 5‐year OS in the training cohort, respectively; (E‐G) The calibration plots for predicting 1‐year, 3‐year, and 5‐year OS in the validation cohort, respectively; (H) ROC curves in the training cohort; (I) ROC curves in the validation cohort. OS, overall survival; ROC, Receiver operating characteristic

A prognostic nomogram was further established for OS via integrating SRXN1 expression and other independent prognostic factors identified in the multivariate analysis. We could use the total score, which was calculated by adding up each point of each parameter, to predict the survival rate of patients at different time after surgery (Figure 7A). The calibration curves were drawn to compare the predicted survival with the observed survival, of which, x‐axes were nomogram‐predicted OS, the y‐axes were actual OS. Good consistency between prediction and observation were shown in the calibration curve for the probabilities of 1‐year, 3‐year, and 5‐year survival (Figure 7B‐D). ROC curves, Harrell's C‐index, and the calibration curves were analyzed to validate the accuracy of the prognostic nomogram. The AUC and C‐index of the nomogram based on SRXN1 expression for OS prediction were 0.693 and 0.672, respectively, which were greater than that of BCLC staging system (AUC:0.593, *P* = .022; C‐index:0.623) and TNM staging system (AUC:0.576, *P* < .001; C‐index:0.596). In addition, with SRXN1 expression replenished for OS, the OS prediction accuracy of both BCLC and TNM staging system were elevated according to the AUC and C‐index (Table 4; Figure 7H and I). In addition, AUC values in the time‐dependent ROC curves showed the nomogram had a good predictive value for the OS of HCC patients in the training cohort (Figure S3A).

**Table 4:** Comparison of the predictive accuracies of nomogram among 2 staging systems according to the AUC (ROC curve) and C‐index

Variables	Overall Survival					
	Training cohort			Validation cohort		
	AUC (95% CI)	*P* value	C‐index (95% CI)	AUC (95% CI)	*P* value	C‐index (95% CI)
SRXN1	0.561(0.490‐0.630)		0.588(0.537‐0.639)	0.688(0.555‐0.820)		0.634(0.552‐0.715)
TNM stage	0.576(0.505‐0.644)		0.596(0.548‐0.644)	0.672(0.543‐0.784)		0.577(0.513‐0.640)
TNM stage + SRXN1	0.612(0.542‐0.679)	.194^a^	0.645(0.587‐0.704)	0.793(0.674‐0.885)	.008^a^	0.663(0.581‐0.744)
BCLC stage	0.593(0.523‐0.661)		0.623(0.570‐0.677)	0.578(0.448‐0.701)		0.539(0.450‐0.627)
BCLC stage + SRXN1	0.608(0.537‐0.675)	.420^a^	0.647(0.586‐0.707)	0.721(0.595‐0.826)	.026^a^	0.648(0.555‐0.740)
Nomogram	0.693(0.625‐0.755)		0.672(0.609‐0.734)	0.817(0.701‐0.903)		0.696(0.613‐0.778)
Nomogram vs TNM stage		<0.001^b^			.004^b^	
Nomogram vs BCLC stage		0.022^b^			<.001^b^	

Abbreviations: AUC, Area Under the Curve; BCLC, Barcelona Clinic Liver Cancer; C‐index, Harrell's concordance index; CI, confidential interval; ROC curve, Receiver operating characteristic curve; TNM, Tumor‐Nodes‐Metastases.

^a^ Comparison of the AUC of the original model with or without SRXN1 expression.

^b^ Comparison of the AUC of the nomogram with TNM stage and BCLC stage in patients with HCC.

In the validation cohort, similarly, the calibration plot presented an optimal agreement between prediction and actual observation in the probabilities of 1‐year, 3‐year, and 5‐year survival (Figure 7E‐G). The AUC and C‐index of the nomogram for OS prediction were 0.817 and 0.696, respectively, which were also higher than that of BCLC staging system (AUC:0.578, *P* < .001; C‐index:0.539) and TNM staging system (AUC:0.672, *P* = .004; C‐index:0.577). The AUC and C‐index of BCLC staging system also improved after SRXN1 expression replenishing for OS (AUC:0.721; C‐index:0.648), as well as the TNM staging system (AUC:0.793; C‐index:0.663) (Table 4). The time‐dependent ROC curves also showed the nomogram had a good OS predictive value for HCC patients (Figure S3B). In summary, the above results indicated that SRXN1 might be a candidate biomarker for predicting the prognosis of HCC patients.

### 3.6 Network analysis of SRXN1 and its associated genes

**Figure 8:**
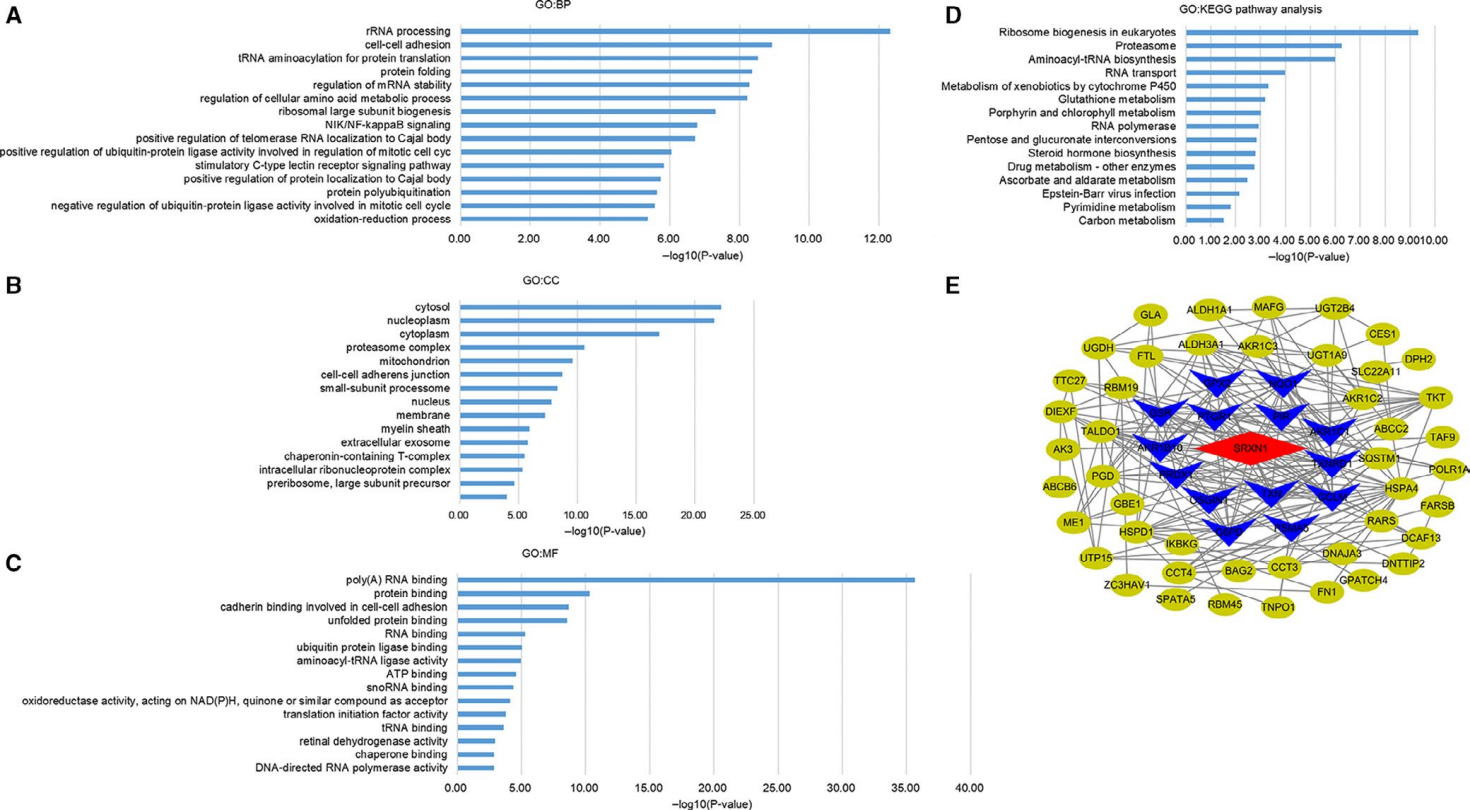
Functional and pathway enrichment analysis and PPI network analysis of SRXN1 and its related genes. (A) Biological processes (BP) analysis; (B) Cell component (CC) analysis; (C) Molecular function (MF) analysis; (D) KEGG pathway analysis; (E) Protein‐protein interaction network. Those genes in navy blue were the first neighbors of SRXN1

A total of 651 positively genes correlated with SRXN1 were found in UALCAN website using a Pearson correlation coefficient (PCC) of 0.3. However, none negatively genes correlated with SRXN1 were discovered, with their PCC value lower than 0.3. Then online software DAVID was used to explore the GO function and pathway enrichment analysis of SRXN1 and its associated genes. GO biological processes (BP) showed that SRXN1 and its related genes were significantly associated with rRNA processing and cell‐cell adhesion, while GO cell component (CC) stated that the 652 genes were markedly enriched in cytosol and nucleoplasm. For molecular function (MF), 652 genes significantly participated in poly(A) RNA binding and protein binding. In addition, KEGG pathway analysis showed the most significantly enriched pathways were ribosome biogenesis in eukaryotes, proteasome, aminoacyl‐tRNA biosynthesis, RNA transport, and metabolism of xenobiotics by cytochrome P450 (Figure 8A‐D). We constructed a PPI network of SRXN1 and its associated genes using online software STRING and software Cytoscape. The result showed thioredoxin reductase 1(TXNRD1), NAD(P)H quinone dehydrogenase 1(NQO1), oxidative stress‐induced growth inhibitor 1(OSGIN1), glucose‐6‐phosphate dehydrogenase (G6PD), glutamate‐cysteine ligase modifier subunit (GCLM), thioredoxin (TXN), PRDX1, glutathione‐disulfide reductase (GSR), pirin (PIR), proteasome 20S subunit alpha 5(PSMA5), prostaglandin reductase 1(PTGR1), glutathione peroxidase 2(GPX2), aldo‐keto reductase family 1 member C1(AKR1C1), and aldo‐keto reductase family 1 member B10(AKR1B10) were the first neighbors of SRXN1, which mainly participated in response to oxidative stress (Figure 8E). Studies had revealed that, among those first neighbor genes, TXNRD1,^28^ NQO1,^29^ GPX2,^30^ and PRDX1^31^ were all the prognostic factors of HCC patients. Sorafenib is the first FDA‐approved multikinase inhibitor for HCC, although some patients may develop resistance to it.^32^ Inhibition of TXNRD1 can make HCC more sensitive to sorafenib treatment by increasing ROS production.^33^ SRXN1 and TXNRD1 have the strongest correlation (Pearson CC = 0.88) among all the related genes. Hence, we further investigated the correlation between SRXN1 expression and the response to sorafenib treatment. Interestingly, SRXN1 was overexpressed in sorafenib resistance subgroup compared to sorafenib sensitive subgroup. And, SRXN1 overexpression brought patients worse OS in HCC patients with sorafenib treatment (Figure S4). The above data indicated us that SRXN1 expression might be related to the response to sorafenib treatment for HCC.

## 4 DISCUSSION

ROS are generated as the by‐products of aerobic metabolism or as an intracellular messenger responding to the exogenous sources.^34^ Excessive ROS damages the cells by activating proto‐oncogenes, inactivating tumor suppressor genes, incomplete DNA repair, nitrotyrosine formation, angiogenesis stimulation, and lipid peroxidation, while the low level of ROS promotes cell cycle progression.^35^, ^36^ Additionally, unlike normal cells, cancer cells may suffer intrinsic oxidative stress due to the high level of ROS caused by metabolic abnormalities and oncogenic signaling, which makes them form improved antioxidant capacity to counteract the oxidative stress and maintain themselves to be alive. Antioxidant enzymes, as the name suggests, play a vital role in reducing intracellular ROS, which in turn reduces oxidative stress. Hence, tumor cells strengthen their antioxidant capacity by raising the level of antioxidant enzymes.^37^, ^38^, ^39^, ^40^ Research had suggested SRXN1 inhibitor might cause the death of human lung adenocarcinoma cells by increasing the level of ROS. Moreover, tumorigenic ovarian cells became more susceptible to ROS‐mediated cell death than nontumorigenic cells with SRXN1 inhibitor treatment.^34^ Recent mounting evidence demonstrated that the overexpression of SRXN1 promoted the migration and invasion of multiple types of cancer cells, including cervical cancer,^12^ colorectal cancer cell,^13^ lung cancer,^41^ and melanoma.^42^ Thus, as an antioxidant protein, SRXN1 might undoubtedly contribute to the survival of cancer cells and play an important role in tumorigenesis.

In this study, we found that SRXN1 was overexpressed in the HCC patients. What's more, SRXN1 overexpression presented good power of predicting the outcomes of HCC patients, even patients with alcohol consumption and hepatitis virus infection, or patients with advanced clinicopathological parameters, using samples from both public databases and Zhongshan Hospital. The time‐dependent ROC curves showed SRXN1 had a good biomarker performance for HCC patients. All these reminded us that SRXN1 could serve as a target for HCC treatment. Due to the process of hepatocarcinogenesis is complex, algorithms integrating prognostic factors may better predict patient outcome. Nomograms had been widely used prognostic models and was proved to be more accurate than routine staging systems for predicting prognosis of various cancers.^43^, ^44^, ^45^As a result, we constructed a prognostic nomogram integrating SRXN1 expression and other independent prognostic factor including cirrhosis, HBsAg, and TNM stage. The AUC and C‐index of the nomogram based on SRXN1 expression for OS prediction were greater than that of BCLC and TNM staging system, which indicated us that the nomogram was more accurate than BCLC and TNM staging system for predicting prognosis of HCC. Hence, the nomogram based on SRXN1 expression might be a good prognostic model for HCC patients. Finally, to further investigate the mechanism of SRXN1 as an oncogene, PPI network analysis of SRXN1 and its related genes were conducted. And, we found SRXN1‐related genes are mainly involved in the regulation of redox balance. Nuclear factor E2‐related factor 2 (NRF2) plays an irreplaceable role in maintaining the redox balance. Inhibition of NRF2 expression could increase the sensitivity of HCC cells to sorafenib, as could TXNRD1, a downstream target gene of NRF2.^33^, ^46^ Due to the strong correlation between TXNRD1 and SRXN1, we investigated the relationship between SRXN1 expression and the response to sorafenib treatment for HCC. SRXN1 was overexpressed in sorafenib resistance HCC and SRXN1 overexpression brought HCC patients with sorafenib treatment worse OS. All these suggest that SRXN1 expression might be related to the response to sorafenib treatment for HCC, although more studies are needed.

There are still several limitations to be solved, while SRXN1 revealed good power of predicting outcomes for HCC patients in our study. First, the sample size is limited. More patients will be included for further study. Second, related experiments in vivo and in vitro are needed to explore the role of SRXN1 in the tumorigenesis and progression of HCC, as well as the mechanism. Third, more studies are needed to investigate the relationship between SRXN1 expression and the response to sorafenib treatment for HCC.

## 5 CONCLUSION

SRXN1 was overexpressed in HCC patients and could serve as an independently prognostic biomarker for HCC. Meanwhile, our study indicated that SRXN1 might play a pivotal role in the tumorigenesis and progression of HCC and could predict outcomes of HCC patients by being integrated to the current nomogram model, which might guide the clinical therapies. However, further studies are needed to confirm the role and the underlying mechanism of SRXN1 in HCC.

## CONFLICT OF INTERESTS

The authors declare that they have no competing interests.

## AUTHOR CONTRIBUTIONS

XJL and YBW conceived and supervised the project. SJL downloaded and performed the bioinformatics analyses. YBW provided the clinical samples. YFF and QF performed the experiments. RQW, ZSL, and GMZ performed the statistical analyses and wrote the manuscript. WLS revised the article. All authors read and approved the final manuscript.

## ETHICAL APPROVAL

All the tumor and paracancerous tissue samples were obtained based on the informed consent of the patients and our study was approved by the ethics committee of Zhongshan Hospital.

## Supporting information

Figure S1

Figure S2

Figure S3

Figure S4

Table S1

## Data Availability

All data generated or analyzed during this study are included in this published article and its supplementary information files.
