# Supplementary figures and images for: Sulfiredoxin‐1 is a promising novel prognostic biomarker for hepatocellular carcinoma
Source: Cancer Med. 2020 Sep 21;9(22):8318–32. doi: 10.1002/cam4.3430 (PMC7666720; doi:10.1002/cam4.3430)

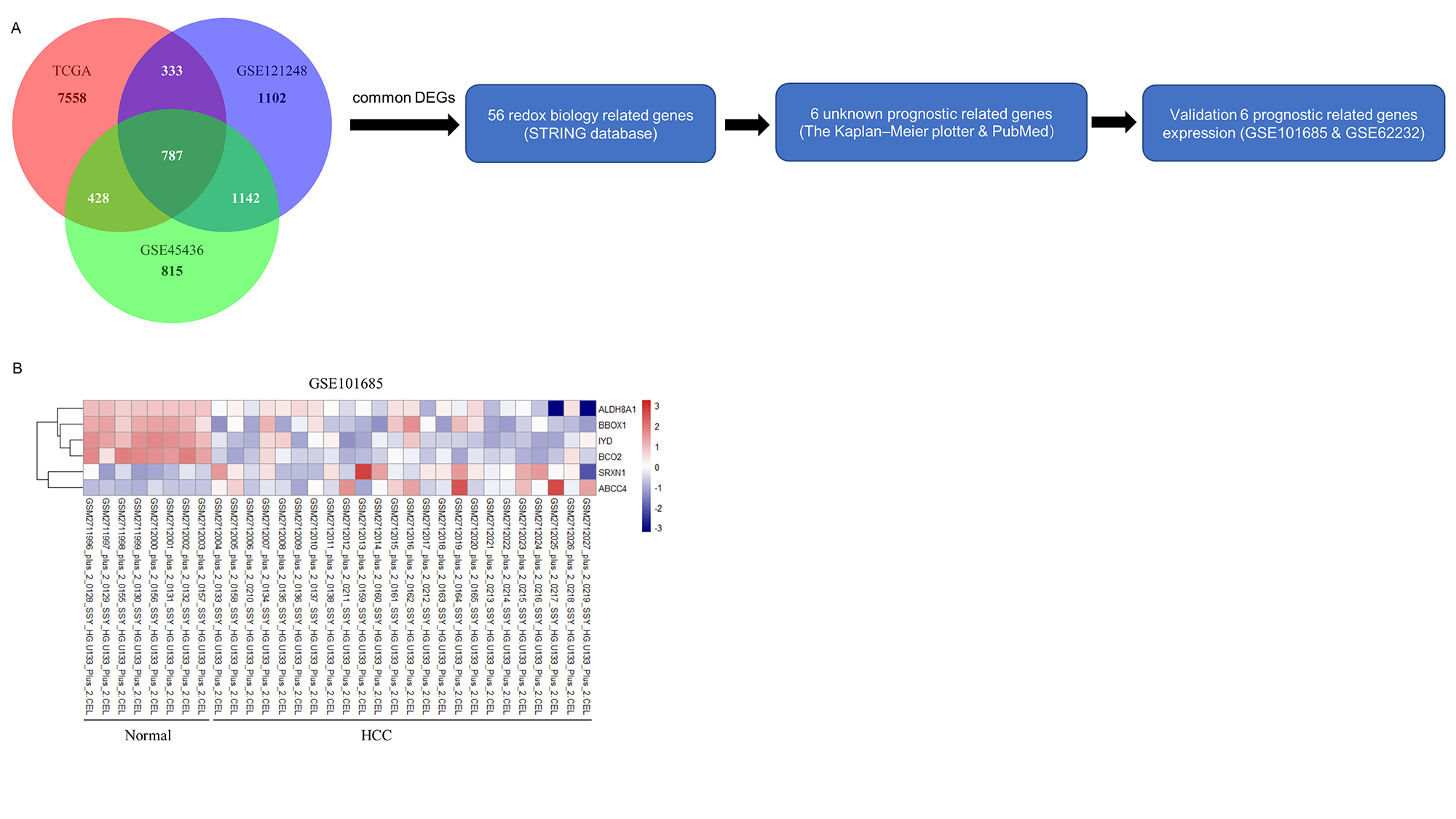

Supplement: Supplementary file 1 — Figure S1 [file CAM4-9-8318-s001.tif]

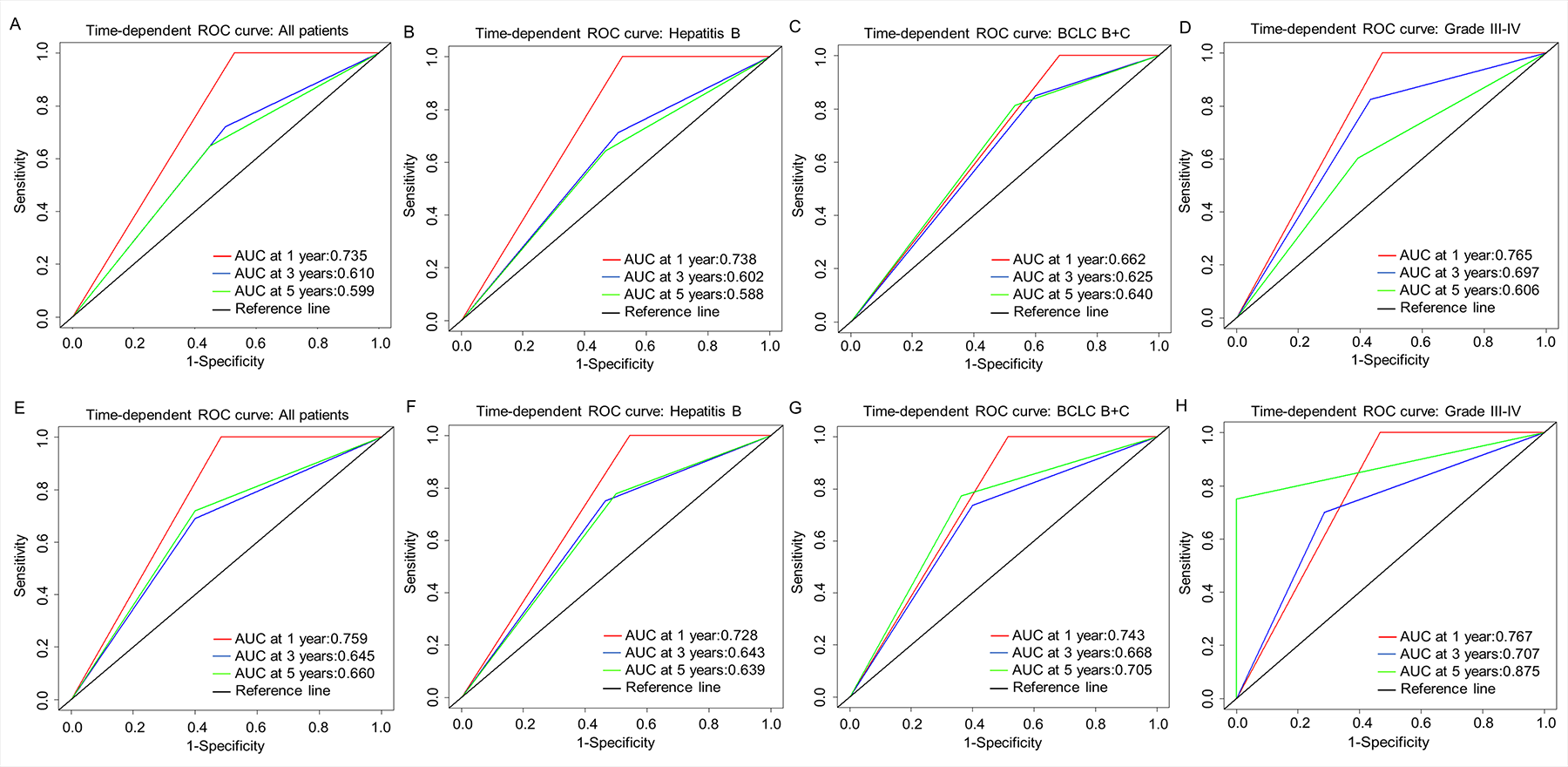

Supplement: Supplementary file 2 — Figure S2 [file CAM4-9-8318-s002.tif]

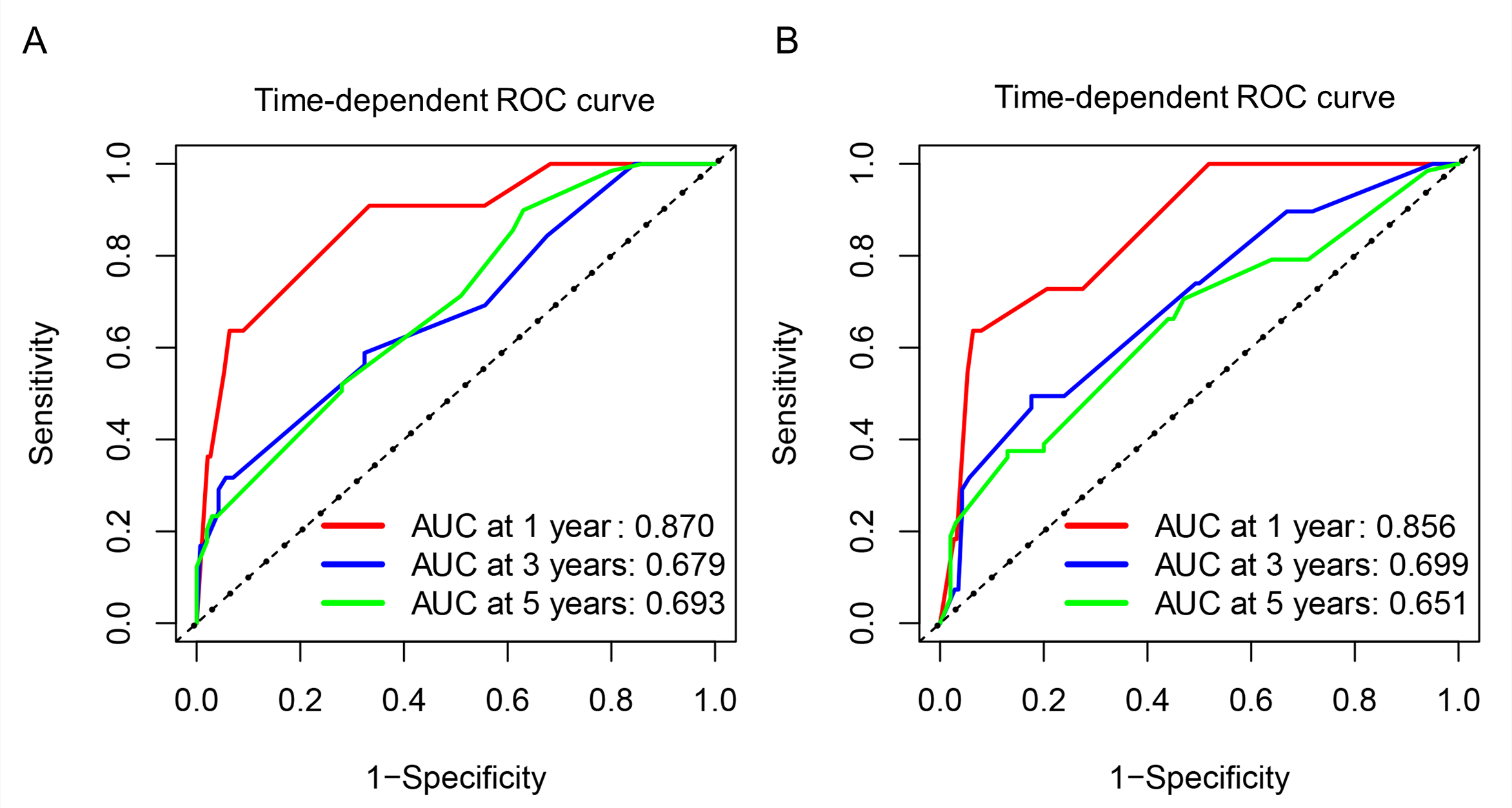

Supplement: Supplementary file 3 — Figure S3 [file CAM4-9-8318-s003.tif]

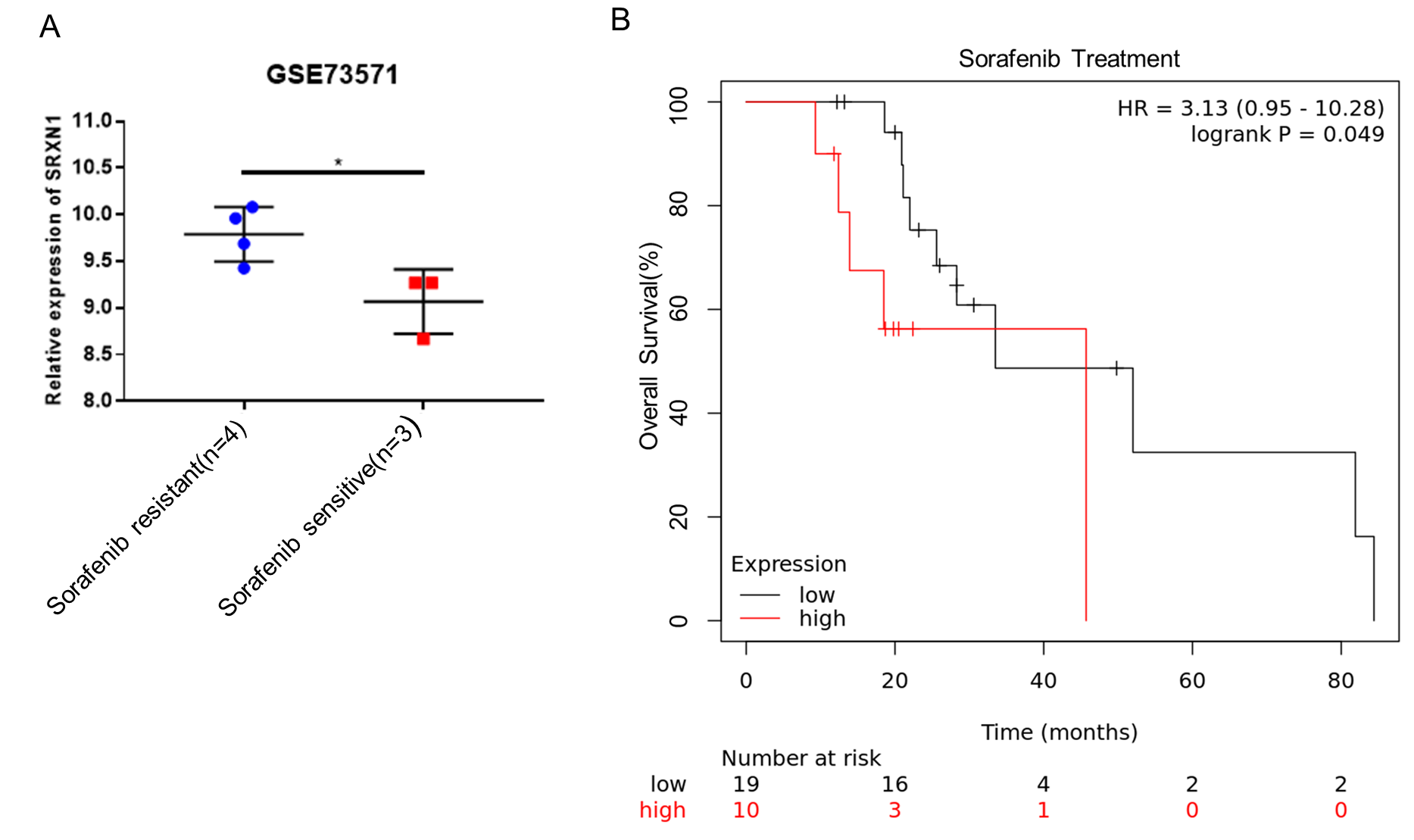

Supplement: Supplementary file 4 — Figure S4 [file CAM4-9-8318-s004.tif]
