# Supplementary material for: Sulfiredoxin‐1 is a promising novel prognostic biomarker for hepatocellular carcinoma
Source: Cancer Med. 2020 Sep 21;9(22):8318–32. doi: 10.1002/cam4.3430 (PMC7666720; doi:10.1002/cam4.3430)
Supplement: Supplementary file 5 — Table S1 [file CAM4-9-8318-s005.docx]

**Table S1 The Expression of SRXN1 in HCC based on the IHC scoring**

| **Expression of SRXN1** | **Training cohort(N=205)** | | **Validation cohort(N=64)** | |
| --- | --- | --- | --- | --- |
|  | **Numbers** | **%** | **Numbers** | **%** |
| Strong | 51 | 24.88 | 16 | 25.00 |
| Moderate | 65 | 31.71 | 18 | 28.13 |
| Weak | 57 | 27.80 | 26 | 40.63 |
| Negative | 32 | 15.61 | 4 | 6.25 |
| High expression | 116 | 56.59 | 34 | 53.13 |
| Low expression | 89 | 43.41 | 30 | 46.89 |

Abbreviation: IHC, Immunohistochemistry.
